# Supplementary material for: Ag-Activated Metal−Organic Framework with Peroxidase-like Activity Synergistic Ag+ Release for Safe Bacterial Eradication and Wound Healing
Source: Nanomaterials (Basel). 2022 Nov 17;12(22):4058. doi: 10.3390/nano12224058 (PMC9696893; doi:10.3390/nano12224058)
Supplement: Supplementary file 1 [file nanomaterials-12-04058-s001.zip › nanomaterials-1992398-supplementary.pdf]

nanomaterials

Supporting Information

Ag-Activated Metal–Organic Framework with Peroxidase-like Activity Synergistic Ag^+ Release for Safe Bacterial Eradication and Wound Healing

Jie Zhou [1,2,†], Ning Chen [3,4,†], Jing Liao [2,3,†], Gan Tian [5], Linqiang Mei [2], Guoping Yang [1,*], Qiang Wang [3,*] and Wenyan Yin [2,*]

[1] Jiangxi Key Laboratory for Mass Spectrometry and Instrumentation, Jiangxi Province Key Laboratory of Synthetic Chemistry, East China University of Technology, Nanchang 330013, China
[2] CAS Key Laboratory for Biomedical Effects of Nanomaterials and Nanosafety, Institute of High Energy Physics, Chinese Academy of Sciences, Beijing 100049, China
[3] Laboratory for Micro-Sized Functional Materials, Department of Chemistry and College of Elementary Education, Capital Normal University, Beijing 100048, China
[4] School of Energy and Chemical Engineering, Ulsan National Institute of Science and Technology (UNIST), Ulsan 44919, Republic of Korea
[5] Institute of Pathology, The First Affiliated Hospital, Third Military Medical University, Chongqing 400038, China
* Correspondence: erick@ecut.edu.cn (G.Y.); qwchem@gmail.com (Q.W.); yinwy@ihep.ac.cn (W.Y.); Tel.: +86-18800173887 (G.Y.); +86-13811227118 (Q.W.); +86-18511550963 (W.Y.)
† These authors contributed equally to this work.

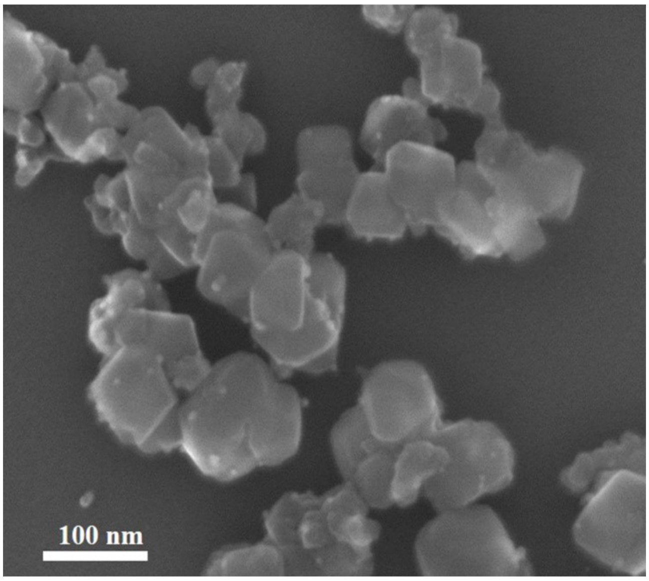

Figure S1. FE-SEM image of UiO-66-NH_2-Ag nanocomposite.

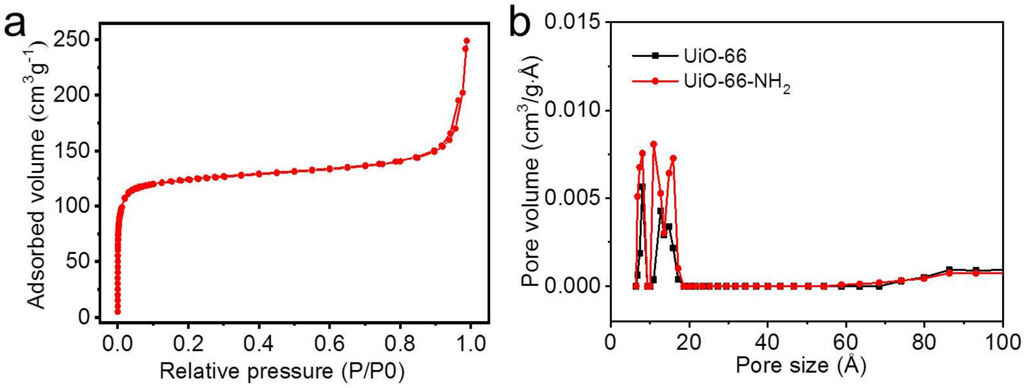

Figure S2. (**a**) N_2 adsorption/desorption isotherms of UiO-66-NH_2 and (**b**) BJH pore size distribution curves of UiO-66 and UiO-66-NH_2.

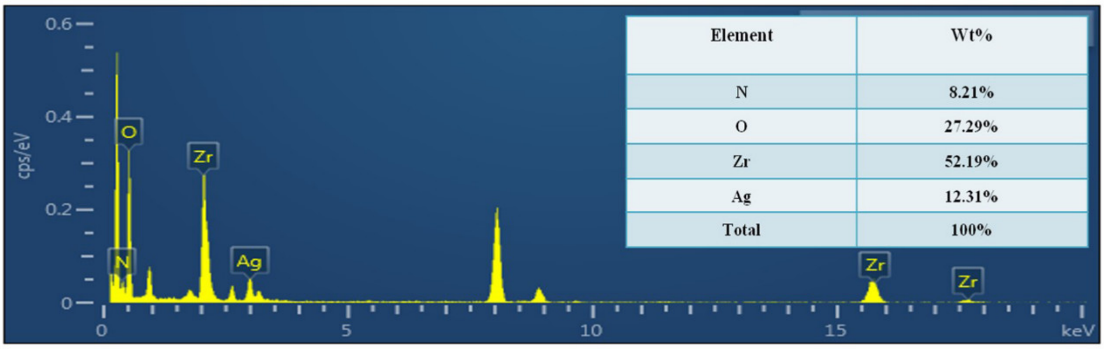

Element	Wt%
N	8.21%
O	27.29%
Zr	52.19%
Ag	12.31%
Total	100%

Figure S3. EDX of UiO-66-NH_2-Ag nanocomposite. Inset is the mass ratio of different elements.

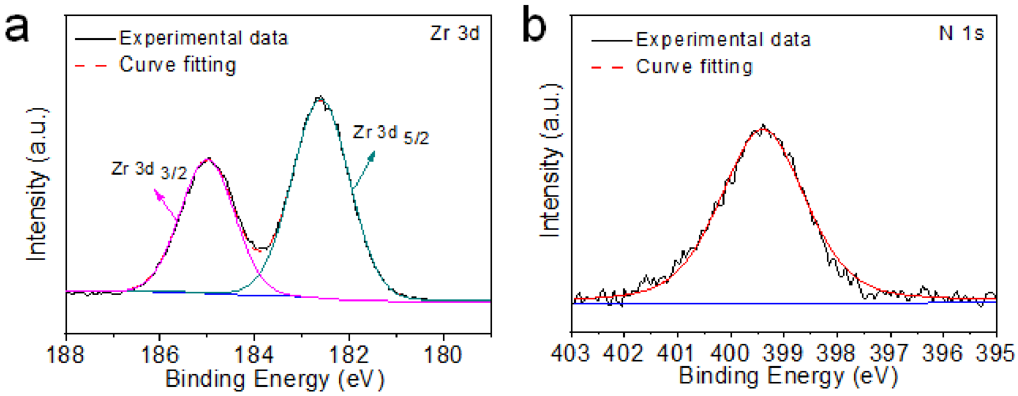

Figure S4. XPS data of high-resolution (**a**) Zr 3d and (**b**) N 1s of UiO-66-NH_2-Ag nanocomposite.

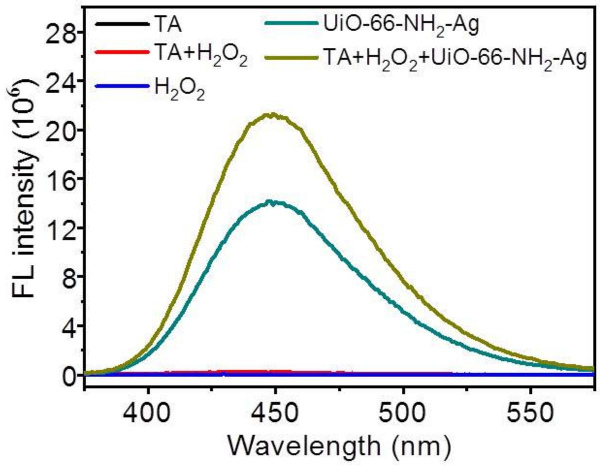

Figure S5. FL spectra of TAOH after different treatments.

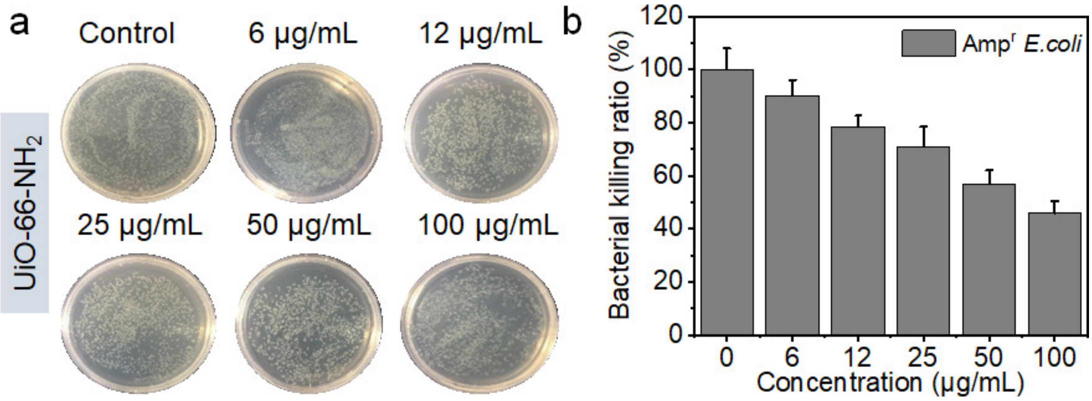

Figure S6. (**a**) Photographs of bacterial colonies formed by Amp^r *E. coli* exposed to different concentrations of UiO-66-NH_2. (**b**) Amp^r *E. coli* killing ratio treated with different concentrations of UiO-66-NH_2.

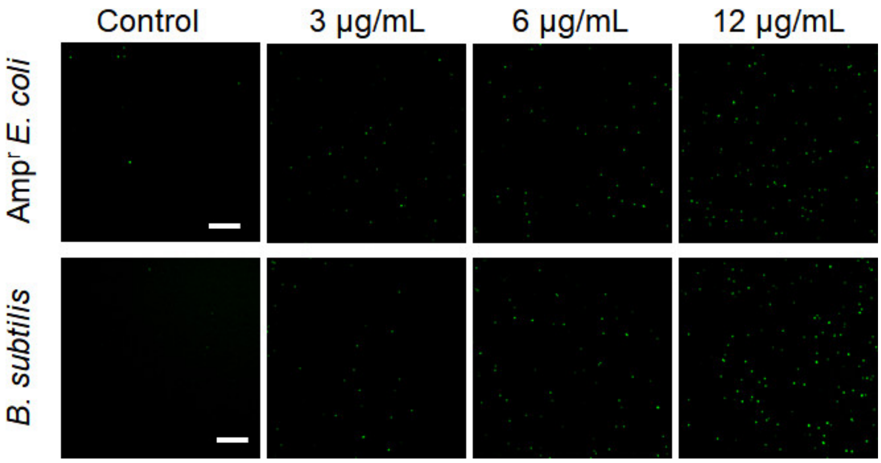

Figure S7. Fluorescence images of Amp^r *E. coli* and *B. subtilis* incubated with different concentrations of UiO-66-NH_2-Ag using DCFH-DA probe for ROS detection. Scale bars: 50 μm.

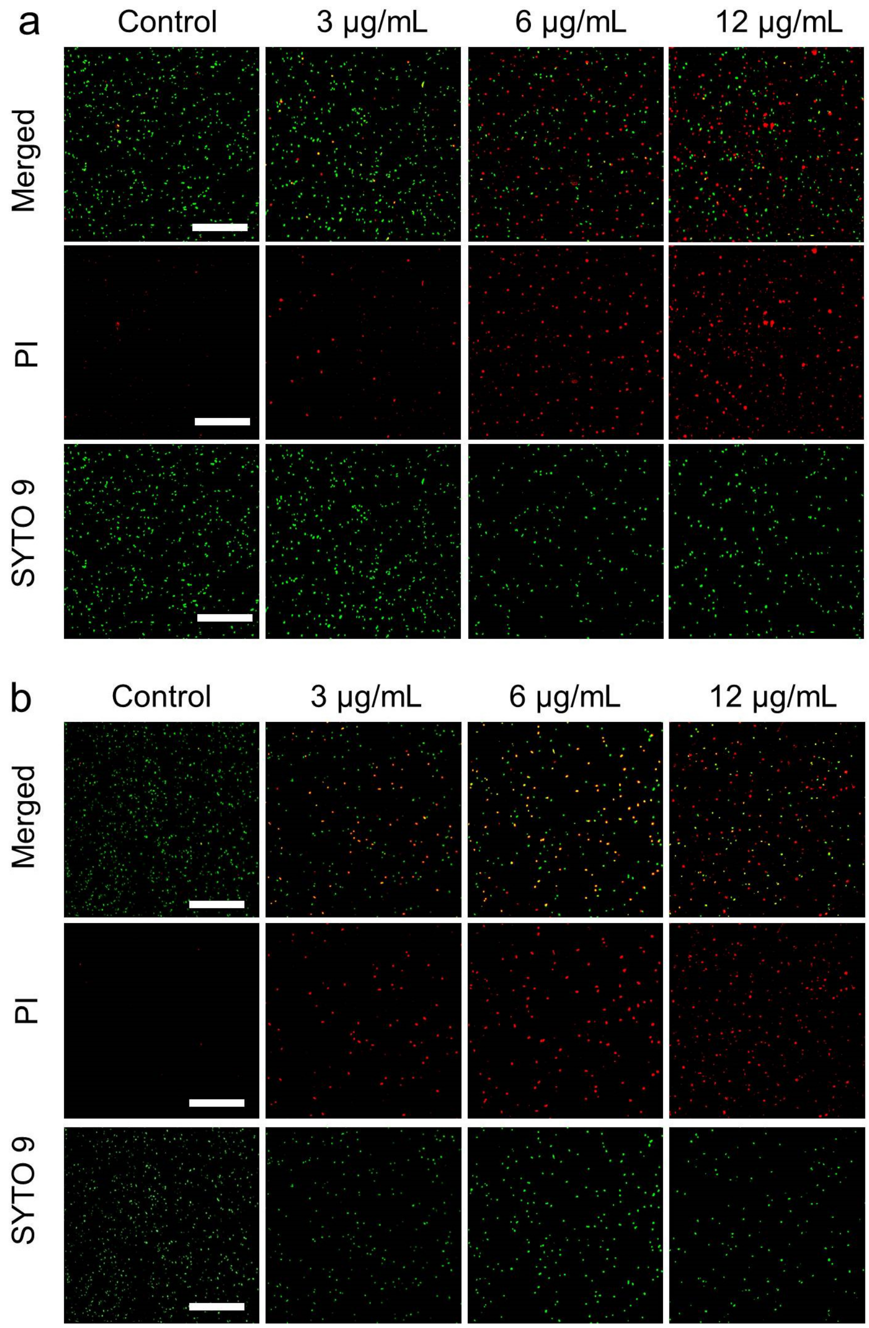

Figure S8. Fluorescence images of (**a**) Amp[r] *E. coli* and (**b**) *B. subtilis* treated with different concentrations of UiO-66-NH_2-Ag incubated with SYTO-9 and PI. Scale bars: 100 μm.

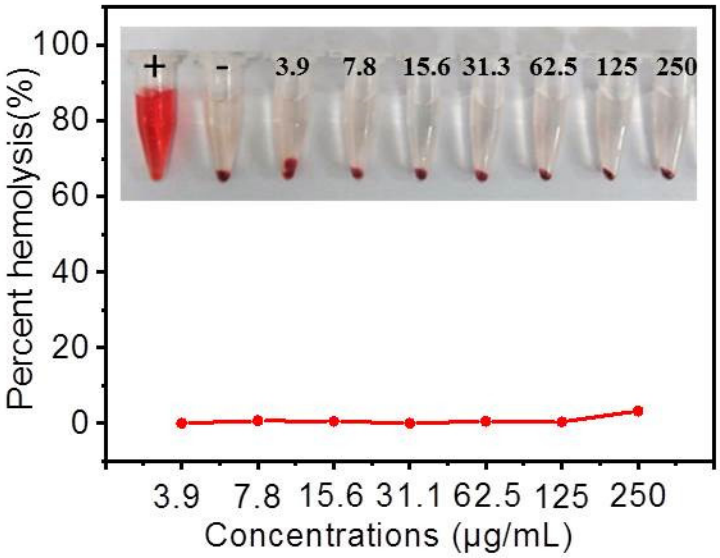

Figure S9. Hemolysis experiment of UiO-66-NH_2-Ag at different concentrations. Inset is the corresponding photograph of the UiO-66-NH_2-Ag at different concentrations.

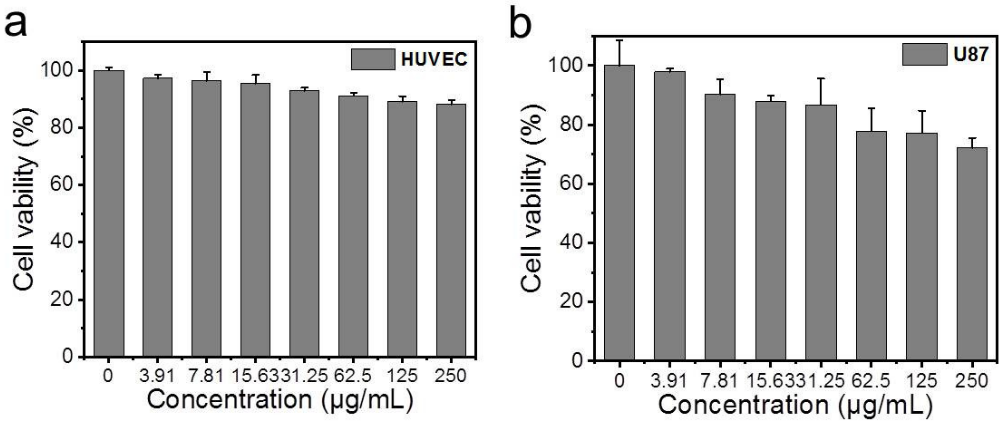

Figure S10. Cell viabilities of (**a**) HUVEC and (**b**) U87 cells after incubation with different concentrations of UiO-66-NH_2 -Ag for 24 h.

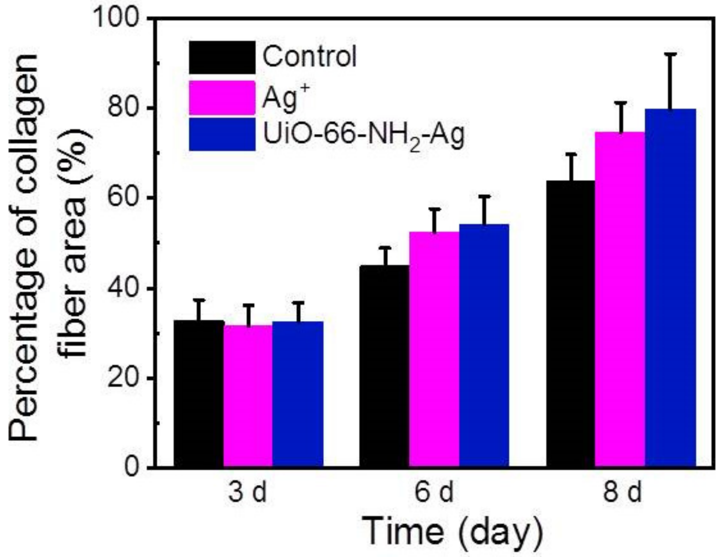

Figure S11. Percentage of collagen fiber area after different treatments during treatment.

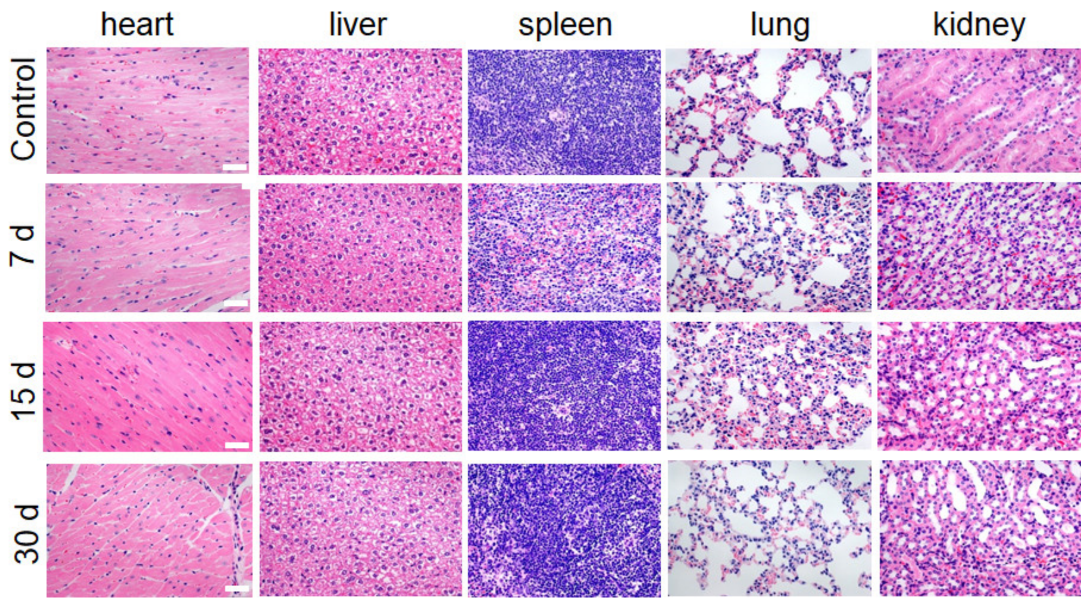

Figure S12. Representative H&E stained images of major organs of mice after treated with UiO-66-NH_2-Ag for different days. Scale bars: 20 μm.

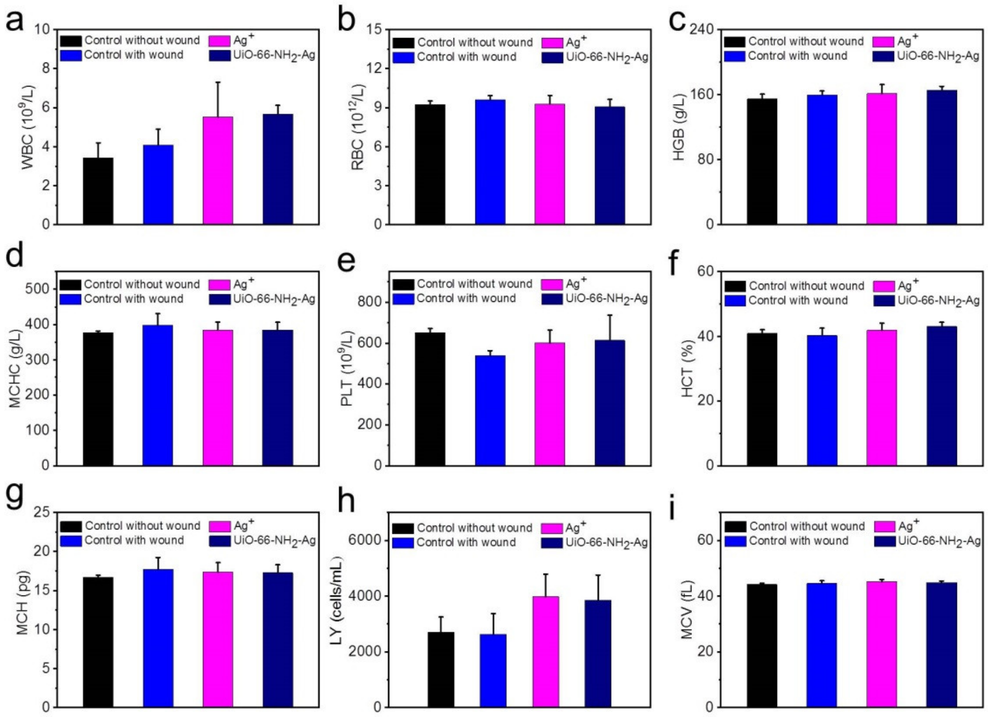

Figure S13. Routine blood analysis of mice after 8 days of treatment. The results show the (**a**) white blood cell (WBC), (**b**) red blood cell (RBC), (**c**) hemoglobin (HGB), (**d**) mean corpuscular hemoglobin concentration (MCHC), (**e**) platelets (PLT), (**f**) hematocrit (HCT), (**g**) mean corpuscular hemoglobin (MCH), (**h**) lymphocyte (LY) and (**i**) mean corpuscular volume (MCV) in each group.
